# A Socioecological Approach to Support the Transition to Adult Care for Youth With Medical Complexity: Family Perspectives and Recommendations

**DOI:** 10.1111/hex.70077

**Published:** 2024-12-29

**Authors:** Lin Li, Nancy Carter, Jan Willem Gorter, Linda Till, Marcy White, Patricia H. Strachan

**Affiliations:** ^1^ School of Nursing McMaster University Hamilton Ontario Canada; ^2^ University Medical Center Utrecht Utrecht The Netherlands; ^3^ Department of Pediatrics McMaster University Hamilton Ontario Canada; ^4^ CanChild Centre for Childhood Disability Research McMaster University Hamilton Ontario Canada

**Keywords:** medical complexity, patient‐oriented research, qualitative, socioecological model, transition to adult care, transition to adulthood, youth

## Abstract

**Introduction:**

The transition from paediatric to adult health care (i.e., ‘health care transition’) poses many challenges for youth with medical complexity (YMC) and their families. YMC need specific approaches to supporting transition, tailored to individual youth and family contexts. In this study, we examine the contextual factors influencing families' transition experiences and describe their recommendations for improving the experience.

**Methods:**

We conducted a qualitative explanatory case study in Ontario, Canada. We completed 21 interviews with 17 participants (11 mothers, 2 fathers, 2 YMC, 2 siblings) from 11 families of YMC. Six YMC (55%) were under 18 years of age (pre‐transfer) and five (45%) were aged 18 years and older (post‐transfer). Analytic approaches included reflexive thematic analysis and directed content analysis.

**Findings:**

Participants described how the interplay of personal and environmental factors impacted their transition experiences. Recommendations for health care providers focused on providing instrumental and psychological support, advocacy and care continuity. Families expressed a need for better access to information and support from primary care providers. System‐level recommendations included streamlining transition processes, improving adult health care services and expanding community supports. A socioecological model is presented to guide health care providers and decision makers in assessing and tackling the challenges faced by YMC and their families during transition.

**Conclusion:**

Findings highlight the complexity and scope of issues surrounding the transition to adult care for YMC in Ontario, with evidence of major gaps in services across multiple sectors and settings. Ongoing efforts are needed to move evidence into practice and advocate for more equitable and responsive care for YMC during the transition and beyond.

**Patient or Public Contribution:**

The research team included two parent co‐researchers with lived experience, who contributed to protocol refinement, funding acquisition, recruitment, findings interpretation and ongoing knowledge translation efforts.

## 1 Introduction

Health care transition (HCT) is the process of moving from a child‐centred to adult‐focused model of health care [1]. This transition goes beyond the medical ‘transfer’ of care and impacts many aspects of the youth's and family's lives (e.g., education, employment, relationships) [2, 3]. HCT is often marked by significant changes in care structures, support systems and overall life trajectories, leaving youth susceptible to fragmented or inadequate care [1, 3, 4, 5, 6]. Poorly supported transitions can lead to gaps in care and negative health outcomes [6, 7, 8, 9, 10, 11], as well as psychological and financial stress for families [5, 12, 13, 14, 15].

While HCT is difficult for most youth with special health care needs, it is particularly challenging for youth with medical complexity (YMC) [2, 16, 17], a subset of young people with chronic conditions who have significant functional limitations, extensive caregiving needs and high health resource utilization [18]. YMC are a heterogeneous group, with a wide range of chronic health conditions, such as cerebral palsy, muscular dystrophy, acquired brain injury, multisystem disorders and life‐limiting or life‐threatening conditions. Medical advances have contributed to rapidly increasing numbers of YMC reaching transition age and transferring to adult health care systems that are ill‐prepared to meet their needs [19, 20, 21, 22]. As YMC are often medically fragile and dependent on medical technology, HCT, and the potential for care discontinuity, poses a significant health risk for this group [23]. This risk is compounded by the large number of health care services that YMC rely on [24], most of which will change during HCT and some of which will fail to be replaced altogether [13, 23, 25].

To date, interventions to support HCT have largely focused on enhancing youth and caregiver knowledge, self‐advocacy and self‐management skills [26]. However, as family caregivers already provide most of the YMC's care [27]—and will continue to do so into adulthood—these interventions have limited applicability for this population [28]. Due to the unique and extensive care needs of this group and the associated burden on caregivers, YMC and their families urgently need support before, during and after HCT to ensure high‐quality care and to improve transition outcomes of YMC and their families [29].

As the youth's and family's interactions with their environment play an important role in their health and well‐being [30], an understanding of how a family's context influences their transition experiences is essential for tailoring this support. Furthermore, while previous studies involving similar populations have explored general youth and family transition experiences, and barriers and facilitators of HCT [5, 12, 31, 32, 33, 34], few have directly elicited families' recommendations for improving the process. Considering these gaps, our study examines (i) how contextual factors interact to influence HCT experiences of families of YMC and (ii) family recommendations on supporting HCT.

## 2 Conceptual Frameworks

In this study, contextual factors are interpreted using two complementary conceptual frameworks/models: the International Classification of Functioning, Disability, and Health (ICF) [30] and Bronfenbrenner's bioecological model of human development [35, 36]. In the ICF, contextual factors include influences on functioning and disability that are both internal (*personal factors*) and external (*environmental factors*) to the person [30]. Environmental factors are further subdivided into *individual factors*, stemming from the immediate environment of the person, and *societal factors*, or social structures, services and systems that impact the person.

Bronfenbrenner's bioecological model of human development describes *context* as a set of nested subsystems. The *micro‐* and *mesosystems* encompass interactions within the individual's immediate environment, such as interactions with or between family members and care providers. The *exo‐ and macrosystems* represent indirect influences on the individual, such as health policy, health care and education systems and sociocultural environments. The micro‐ and mesosystems correspond with *individual factors* in the ICF, while the exo‐ and macrosystems map onto *societal factors*.

We chose to use the simpler taxonomy of the ICF to categorize contextual factors while drawing from the bioecological model's nested subsystems for visualization. In this study, *personal factors* included youth and family factors, while *environmental factors* included the family's social network and broader societal factors. Additionally, the ICF components of health condition, and body function and structures, were included under youth factors.

## 3 Methods

### 3.1 Study Design

We employed a patient‐oriented, qualitative explanatory case study design. While the study protocol has previously been published [37], a brief overview of the study's methods is provided here. Additional details about patient and public involvement that were previously unpublished are also described.

This work is part of a larger study exploring the experiences of families of YMC during the transition to adulthood. A previous publication reports findings on families' priorities for the youth's future, the challenges they faced, and the process by which they survived and adapted to transition [38]. In this article, we delve deeper into the nuances of how contextual factors influenced these experiences and recommendations from families on improving the HCT process. Two theoretical propositions or ‘hypotheses’ guided the research aims and findings described in this article:

1. Unique combinations of contextual factors will have nonlinear, synergistic and counteractive effects on how families manage the transition.
2. Family recommendations for support will reflect aspects of transition that families find most challenging.

### 3.2 Setting, Participants and Sample

Eligible participants included (i) English‐speaking YMC or their family members, (ii) aged 16 years or older, (iii) residing in Ontario, Canada and (iv) with direct experience of the YMC's transition to adulthood. We defined medical complexity using criteria based on the Provincial Council for Maternal and Child Health's (PCMCH) standard operational definition for children with medical complexity [39]. Specifically, we identified YMC as individuals between the ages of 16 and 30 years, who met the following three out of five PCMCH criteria: (i) chronicity: has a health condition expected to last into adulthood; (2) care intensity: dependent on medical technology for vital bodily functions, requires daily or near‐daily nursing care, or completely physically dependent on caregivers for activities of daily living and (3) complexity: involvement of five or more health care providers/teams while in the child health care system.

In case studies, the desired sample size is based on the unit of analysis [40], which in this study, was the family unit. We sought a purposive sample of 10–15 families (1–3 participants per family). To ensure diverse experiences, we used maximum variation sampling [41], capturing contexts such as urban, rural, pre‐health care transfer (YMC under age 18) and post‐health care transfer (YMC aged 18 and older).

Recruitment primarily occurred through social media targeting online parent communities. Potential participants were asked to contact the primary author, who assessed eligibility based on participant self‐report. Once eligibility was confirmed, the primary author explained the study details and completed the informed consent process electronically. Medical complexity was further verified through participants' descriptions of the youth's care needs during the interviews.

### 3.3 Data Collection and Analysis

Using Zoom videoconferencing software, the primary author conducted and recorded semi‐structured interviews, which were then transcribed verbatim. To allow time for in‐depth exploration, we gave participants the option to split the interview into two sessions or participate in a repeat interview to address new questions. We also asked participants to share resources that supported their HCT (e.g., printed materials, websites), which we categorized as documentary evidence.

We used reflexive thematic analysis for the inductive coding of interview transcripts [42] and directed content analysis for documentary evidence [43]. L.L. and P.S. performed a primary analysis in NVivo 12, and the remaining authors contributed to refining and validating interpretations. L.L. also applied the initial theoretical propositions to (i) deductively interrogate the data; (ii) validate initial themes; (iii) ensure study aims and research questions were adequately addressed and (iv) generate new insights on our initial hypotheses. As each family in this study constituted an ‘embedded’ unit of analysis, we analysed the data within, and then across, the family units. We accomplished this by first coding all data collected, generating a case description for each family, and then examining contextual differences across family units. Additional methodological and quality details are provided in File S1: Consolidated Criteria for Reporting Qualitative Studies (COREQ) Checklist [44] and Strategies to Enhance Rigour.

### 3.4 Patient and Public Involvement

We consulted a variety of patient engagement frameworks in the development of a patient and public involvement plan [45, 46, 47]. We recruited two parent co‐researchers with lived experience through the research team's contacts in the family engagement in research community. In keeping with recommendations from the patient engagement literature [47, 48], the primary author provided onboarding, which involved (i) relationship building; (ii) orientation to the research process; and (iii) clarification of roles, expectations and timelines.

We presented parent co‐researchers with an engagement matrix that described potential roles (consult, involve, collaborate, lead/support) for each project stage (preparation, execution, implementation). We adapted this matrix from Smits et al.'s ‘Involvement Matrix’ [49] and Manafò et al.'s ‘Levels of Patient and Researcher Engagement in Health Research’ [50]. We also provided a list of specific tasks, co‐developed with the parent co‐researchers, with responsibilities, timelines, estimated time commitment and compensation. Parent co‐researchers used the engagement matrix and task list to negotiate their involvement throughout the study.

In the preparation stage, parent co‐researchers contributed to protocol refinement, study procedures and funding acquisition. For example, their input influenced decisions on defining medical complexity, sampling and eligibility criteria, and methods for recruitment and data collection. In the execution stage, they facilitated access to parent communities, optimizing recruitment. Their involvement in the implementation stage is ongoing, and thus far, has included: feedback on findings and lay summaries, co‐presentations, co‐authored publications and dissemination within various communities. Compensation was provided based on established guidelines [51]. The engagement matrix and task list are available in the published protocol [37].

## 4 Findings

### 4.1 Participants

Seventeen participants from 11 families completed 21 interviews between March and August 2021. Participant characteristics have been previously published [38], with a brief overview provided here. Of the 17 participants, there were 11 mothers, 2 YMC, 2 fathers and 2 siblings (1 brother, 1 sister). YMC ages ranged from 16 to 28 years, with six (55%) YMC under 18 years of age (pre‐transfer) and five (45%) YMC aged 18 and older (post‐transfer). Seven (64%) YMC were female and four (36%) were male. Eight (73%) out of 11 families were two‐parent (married) households, eight (73%) had multiple children and seven (64%) lived in urban areas. Annual household income ranged from less than $25,000 (*n* = 3, 27%) to $150,000–200,000 (*n* = 1, 9%). Twelve (92%) of the 13 caregiver participants had completed some form of postsecondary education.

### 4.2 Contextual Factors

Each family's HCT experiences were uniquely shaped by personal and environmental factors. While some factors had clear positive or negative effects, the interaction of multiple factors ultimately determined how families coped with HCT. Thus, while the following discussion is organized into levels of contextual factors, these groupings are fluid, with each section drawing on others. Participant quotes supporting the influence of contextual factors can be found in Table 1.

**Table 1:** Participant quotes supporting the influence of contextual factors.

Domain	Subcategory	Supporting quotes
Youth factors	Health stability	My son's condition is different and it's a progressive changing condition…because we've had so many different doctors and so many different moving parts, it's just harder and more challenging, I guess, just to try to make sure that nothing falls off. (Mother H) I mean, not a whole lot should change once she's 18 and she's through puberty. (Mother A)
	Care complexity	Well, I think in general, with children with disabilities, it is such a variety of disabilities and range and spectrum, like mild, moderate, severe… I find that hard, because when we're looking at [programs], it's always about the mild one, not the severe one. (Mother E) Their disability does not disappear the day they turn 18, but a lot of the funding and opportunities do. (Mother J)
Family factors	Family structure and strengths	We are a family of two. I adopted [youth] when she was quite young. Her biological family were not coping with her complexity of needs. (Mother J) My oldest son really became a huge advocate for me. He wrote letters. He came to meetings. He was really a good rock in all this. (Mother C)
	Socioeconomic status	The stability that my husband's been able, even during COVID, to work and support us and we can, you know, not feel stressed financially. That's huge. (Mother E) When [youth] was still in paediatric panel and in school, I was able to work full time and care for her, do the lion's share of her care. Since she has aged out of school, that has changed. (Mother J)
	Prior experiences	I speak English. I'm familiar with government. I know enough how to fight and how to push. And I will do it. I will do it until things change. But you are dealing with families potentially less educated, less experienced and with a language barrier. I mean, how do those families cope? I have no idea. (Mother D)
Family social network	Extended family and friends	We don't really have family to turn to. Friends don't really understand what we're going through. (Mother B) We have tremendous support…I have family. We both have supportive family. (Mother A)
	Health and social care providers	Our paediatric neurologist was one of her solid, like on her team, in her realm, people. And he wrote and phoned three or four adult neurologists before he found one that would accept her. (Mother J) The truth is that when I ask even a doctor, sometimes they don't know, they tell me I should Google or research it myself. It's pretty frustrating. Doctors do not guide us on the transition to adulthood. (Mother B)
	Families with shared experiences	I mean, to be honest, most of the resources that I find helpful are other parents that are dealing with it because they know exactly the struggles they've gone through or the hoops that they've had to jump through. (Mother H)
Societal factors	Regional access to services	Because we're out of region for a lot of the more technical supports that she requires, or the more specialized supports that she requires, it complicated our continued access, or our transition, between some of them. (Mother J) I'm grateful that we live in a rural area…because I think she's so complex compared to the other kids in this area that people are paying attention to that. (Mother A)
	COVID‐19 pandemic	I think the fact that we're in a pandemic and we're working even more than we ever have, the amount of work that needs to be done can be stressful. (Mother I) So we've tried to put this program together [for young adults with disabilities]. It's really…it's coming along, but it's a very rocky start because of everything that we've had to deal with COVID, it's been hard. (Mother K)
	Ableism/stigma	There are all sorts of families in this situation. Treat us fairly and treat our kids fairly. Treat them like respectable citizens. You wouldn't have treated anybody else—who's working, who can talk, who can participate in society alone—in the same way. (Mother D)

#### 4.2.1 Youth Factors

Critical determinants of HCT experiences included the YMC's health stability and care complexity. For instance, one YMC experienced multiple hospitalizations and surgeries preceding HCT, which not only made it more difficult for the family to focus on transition‐related tasks but also shortened their timeline to prepare for HCT. Furthermore, the hospitalizations led to cancelled ‘transition’ appointments, resulting in missed opportunities for HCT preparation.

The nature of the YMC's care complexity and disabilities also significantly influenced the family's capacity to cope with the transition. HCT led to changes in public funding structures for home care and disability support, with some families receiving an overall increase in financial support, while others experienced a decrease or were put on waitlists that resulted in gaps in services. These discrepancies occurred—despite the YMC's care needs remaining the same—and were attributed to how the YMC's disabilities aligned with government policies determining eligibility for supports. As these policies differed between the paediatric and adult systems, HCT often led to a mismatch between the youth and family's needs and the support provided by the government. Furthermore, participants reported that the high complexity of the YMC's care needs made it more difficult to find suitable adult health care providers, recreational day programmes and paid care workers. These examples illustrate how the interaction between the person and the environment (e.g., health policy) can lead to a range of HCT experiences.

#### 4.2.2 Family Factors

Due to the intensive care needs of YMC, the burden and work of preparing for and navigating HCT primarily falls on their caregivers, with other family members also being affected. Family factors that played a major role in HCT included family structure and strengths; socioeconomic status and prior experiences. Among primary caregivers, eight were married, one was divorced with the other parent involved in care and two were solo caregivers. Family structure and relationships interacted with the youth's care needs and external caregiving support to significantly impact socioeconomic status and HCT experiences. For example, some families were able to earn dual incomes due to adequate home care, while other families relied on a single income. Notably, because of their youth's care needs, both solo caregivers in this study were unable to work. In several cases, caregivers reported that the youth's transition to adult services, and the associated loss of home care hours and school, was the catalyst for leaving employment.

Additionally, HCT brought up fears about who would care for the YMC should they outlive their parents. Families with multiple children (*n* = 8) actively considered how the YMC's siblings might take on future caregiving roles, thereby alleviating some of this uncertainty. While these concerns about the youth's future may not be directly related to HCT, families revealed that these fears are accentuated by the myriad of changes that occur across multiple sectors, including the health care system. These changes are intertwined, and thus, cannot be isolated from HCT [3]. Some caregivers also described having strong advocacy skills and professional or volunteer experiences that helped them navigate the health care and social systems. These roles preceded the youth reaching adulthood and were often chosen in response to having a child with extensive medical and caregiving needs.

#### 4.2.3 Family Social Network

The social context surrounding each family included support or lack thereof from people within their social and care networks, including extended family and friends, health and social care providers, and other families with shared experiences. While some participants reported that extended family and friends were valuable sources of psychological support, not all families had access to these social resources. Beyond psychological support, families looked to their health and social care providers for instrumental support (e.g., sharing information, explaining the HCT process, helping with paperwork, fostering connections) but received varying levels of assistance. For example, some participants shared that their paediatric providers advocated for the YMC's needs to be met in the adult system. Others reported that they expected providers to guide them through the HCT process, but instead received little or unhelpful support. The level of support offered by providers, who have the potential to serve as crucial guides and advocates during HCT, can significantly impact the family's ability to navigate the complexities of the HCT process, and access health care and community services in the adult system.

While participants endorsed mixed experiences with service providers, most agreed that other families with shared experiences were the most helpful resources for navigating HCT. These families provided many forms of support, including anticipatory guidance about the HCT process and adult services; resources on topics such as financial support, education, day programmes and housing; psychological support; and a collective voice for advocacy and change. The multifaceted support provided by other families highlights the need to address transition more broadly across multiple sectors.

#### 4.2.4 Societal Factors

Societal factors that impacted families' transition experiences included regional access to services, the COVID‐19 pandemic and the influence of ableism on health care and social infrastructure. The family's place of residence was a significant determinant of their HCT experiences, as regional services and supports differed greatly across the province. A few participants shared that living in a small, rural community was beneficial, as they felt well supported by their close‐knit community. They also suggested that their rural location contributed to the YMC receiving more attention from government agencies, as they were only one of a few ‘high needs’ individuals in their region. Others described hurdles to accessing care caused by different geographical catchment boundaries for paediatric and adult health care services. Because of inconsistent access to specialists, for some YMC, HCT occurred not only across the paediatric–adult divide but also across geographical regions.

Furthermore, as this study was conducted in 2021, the COVID‐19 pandemic inevitably influenced and changed how families experienced HCT. The pandemic added additional stress and demands to an already overwhelming transition process. It negatively impacted access to home care and made it harder for youth to engage socially with others. The pandemic also forced youth and their families to change their goals for adulthood, such as plans for education, housing and social participation. Some participants reported that due to the scarcity of nurses worsened by the pandemic, they were unable to find hired caregivers to fill their allotted home care hours. Families were unable to use government funding designated for nursing care to hire non‐nursing workers, despite being unable to find nurses to fill their care needs. As a result, families were often left with no caregiving support at all.

Lastly, the sociocultural context—particularly stigma towards disability within the adult systems—was associated with negative transition experiences. Participants attributed many of the injustices they faced (e.g., inadequate access to health care, housing and social programming) to deeply embedded ableism within societal structures. They shared that these injustices were worsened by both the transition to adult services and the COVID‐19 pandemic.

### 4.3 Family Recommendations to Support Health Care Transition

Due to the critical role that families play in the YMC's care, it is paramount that they are consulted in the development and delivery of services to support this population. Families shared their recommendations for health care providers, tools and resources and decision makers to better support HCT. See Table 2 for participant quotes supporting these recommendations.

**Table 2:** Participant quotes supporting family recommendations.

Domain	Subcategory	Supporting quotes
Recommendations for health care providers	Instrumental support	I'd just like to have a guide. I'd like to have a professional that tells us, ‘Ok, this is what's coming up. This is what to expect.’ And to help us know as opposed to having to figure it out on your own. (Father A)
	Psychological support	I think that there is a whole component of psychological support that's missed. I think care providers just don't acknowledge or aren't aware that this is a major transition for the parents psychologically. (Mother A)
	Involvement of primary care providers	I feel that there really needs to be early involvement of a family physician, and I wish I knew that when she was younger, because I felt like there was no need to have a family physician because there was a paediatrician. (Mother H) I think part of the problem in gaining access to the adult specialists, too, was the lack of knowledge in the paediatric world of who's even in the adult world to refer to. And that's where a family physician can be invaluable as that patient transitions into the adult world. (Mother F)
	Advocacy and holistic care	We need more advocacy from nurses and other health professionals and physicians. You know, for their patients. (Mother F) Yes, you're taking physical and medical care of the client, but are you doing meaningful activities with them?… it shouldn't be so clinical in its care. It should be more holistic. (Mother C)
	Care continuity	There should be some checks and balances so that people don't fall off the radar. (Mother H) Setting up like a transfer of care, a team meeting. And even possibly having an overlap of about a year. (Mother F)
Tools and resources	Central source for information	A central place, say online or somewhere, where you can get the source of information and services…Some place you go to instead of having to go dig it up yourself, stumble across it yourself, find out maybe a year or two later you could've gotten something in place but didn't know about it. (Father I)
	Roadmaps, guidelines, checklists	A checklist to say, ‘OK, this doctor has contacted us and we have an appointment to see them, and they've received whatever information they need.’…I think would be helpful just to be able to make sure that you haven't missed a doctor that might have been involved in his care. (Mother H) A step‐by‐step booklet early on that's given to parents. (Mother A)
	Regional lists of services	A listing of professionals that, people who work with people with special needs, with cardiac care. Or dentists that deal with people with special needs. (Father I)
Recommendations for decision makers	Transition process	Somehow that whole process needs to be streamlined, so you're not dealing with all sorts of different people from all sorts of different ministries, and ministries having different rules and regulations and things that you need to apply for and get. (Mother D)
	Adult health care	And I think some of it also comes down to how [physicians] are paid…And you can definitely see the difference in the care because of the model of payment. (Mother F) And there should be just a transition team that goes from [Children's Hospital] to another transition that takes over exactly the same as what [Children's Hospital] offered. (Mother G)
	Community supports	Adequate home nursing. Adequate home PSW support. (Mother J) I just wish there was more of a positive outlook, that it was like, ‘You know what, parents? It's going to be OK. You're going to get respite. There's going to be great programs.’ (Mother E) It's not about having a child that's more disabled or less disabled, but, you know, a system where you can state your costs, state what kind of mental and physical energy and the hours you require out of your normal day to look after your child. And what kind of assistance is there for that? (Sibling C)

#### 4.3.1 Recommendations for Health Care Providers

Family recommendations for health care providers focused on: instrumental and psychological support; involvement of primary care providers; advocacy and holistic care; and care continuity. From their paediatric providers, families expressed the need for instrumental support on how to navigate the HCT process and what to expect from adult services. Participants suggested that this support should ideally come from a central contact person (e.g., a service coordinator) who is knowledgeable about the HCT process and can provide individualized support. Beyond guiding them through the bureaucratic processes of HCT, some participants expressed that the emotional aspects of transition are often overlooked by their health care providers. For families, HCT is more than the transfer of services; it is part of a major life change, intricately linked to shifts in family roles, uncertainty about the future and complex emotions, such as guilt and grief. Participants urged providers to acknowledge the emotional aspects of transition and provide psychological support where needed. Participants also suggested that peer support could be an effective option for addressing emotional needs, but they were often unsure how to access this support.

Several participants shared that having a primary care provider who already knew the YMC could help to facilitate connections to adult health care providers. Participants also suggested that their providers need to advocate more strongly for their clients and deliver care using a humanistic, whole‐person approach. Lastly, families wanted assurance that they would receive continuous care, without gaps caused by waiting periods or missed transfers of care. Participants suggested processes to ensure care continuity, such as joint paediatric–adult team meetings or overlaps in paediatric and adult care.

#### 4.3.2 Tools and Resources

Beyond improving the support from individual providers, families need better access to information about HCT. Notably, participants only shared three resources that they found to be helpful in the HCT process. These resources included a website about social services and financial support for adults with developmental disabilities, a booklet on transitioning to an adult long‐term ventilation programme, and a webinar on regional autism services for adults, all in Ontario, Canada. While some participants found these resources to be helpful, the information provided varied in its applicability to individual YMC and only addressed fragments of a complex transition. Overall, it was clear that families of YMC accessed very few formal sources of information that supported HCT. Participants suggested the creation of tools and resources such as a central source for information; transition roadmaps, guidelines, or checklists; and regional lists of adult services, supports and health care providers who are knowledgeable and willing to care for young adults with medical complexity.

#### 4.3.3 Recommendations for Decision Makers

At the health policy and system level, many participants stressed the importance of streamlining the HCT process, improving adult health care services and infrastructure, and expanding access to community supports for young adults with medical complexity. Participants perceived the process for determining eligibility for adult developmental services as overly onerous, and the division of services and funding between government ministries was confusing and inefficient. Regarding the adult health care system, participants suggested that changes be made to physician billing structures to better reflect the care needs of young adults with medical complexity. They compared care experiences under salary‐based and fee‐for‐service payment models and associated the former with higher quality care for YMC. Furthermore, participants emphasized that essential services from the child health care system should carry over to the adult system (e.g., multidisciplinary complex care clinics).

Participants also advocated for the expansion of community‐based supports, including more options and flexibility for housing, home care and day programmes to replace school. They recommended that these supports be provided based on individual youth and family needs, rather than the type and level of disability. Lastly, while participants identified many gaps and inefficiencies in our public systems, some did not believe that structural change was possible. For example, Mother G stated, ‘It's government and you can't change it. So, no, I wouldn't change anything, I guess, because you can't.’ This finding draws attention to the inequitable power structures of the health and social care systems, and the oppression, invisibility and lack of trust that these inequalities foster for these families.

## 5 Discussion

This patient‐oriented qualitative case study investigated HCT for YMC and their families, focusing on the impact of contextual factors and family recommendations for health care providers and decision makers. This study is among the first to explore family‐identified recommendations on improving HCT for YMC [23], and to our knowledge, is the first Canadian study to do so. Families of YMC emphasized the need for instrumental and psychological support, effective advocacy and care continuity from their providers. Families also require more information and resources on HCT, as well as system changes to streamline the process and improve access to adult services.

**Figure 1:**
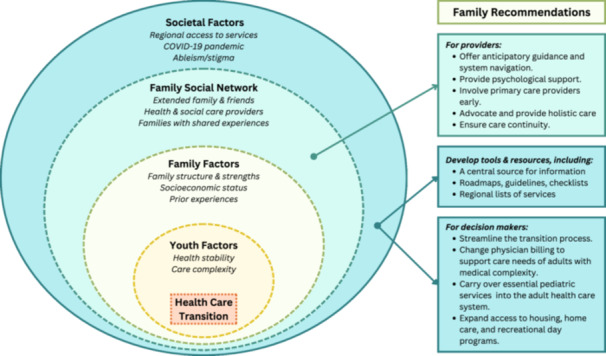
A socioecological model for supporting health care transition for youth with medical complexity. This model illustrates key contextual factors impacting the transition to adult care for youth with medical complexity and their families' recommendations for improving the transition experience.

A conceptual model illustrating the study findings is presented, with relevance for both health care providers and decision makers (Figure 1). Borrowing from the bioecological model [35, 36], personal and environmental factors are depicted as four nested subsystems: youth, family, family social network and societal. However, as HCT is a socially constructed, rather than biological, phenomenon, we have chosen to use the term ‘socioecological’ instead of ‘bioecological’ to describe our model.

Betz and colleagues also proposed an ecological model of HCT for a broader population of youth with special health care needs [52]. Although they identified many similar factors, other variables in the model have limited applicability for YMC. As YMC and their families have different health care needs than other youth, a YMC‐specific HCT model is useful for guiding targeted interventions and policies. Providers can use this model to assess each family's unique context, identify those who may experience more challenging transitions, and provide tailored support. Furthermore, the current health and social care systems are clearly, deeply inadequate at supporting the needs of adults with medical complexity. Thus, these recommendations should be considered by decision makers when designing health care and social policy. These decisions, which greatly impact the quality of life for YMC and their families, should prioritize equitable and safe access to adult care for this group.

### 5.1 Implications for Practice and Policy

An important step in designing future interventions and policies is to determine which relevant factors are modifiable. The personal (youth/family) factors identified in our study were mostly non‐modifiable and too individualized to guide meaningful recommendations for the entire group. On the other hand, many of the environmental factors are modifiable, and those that overlap with family recommendations are optimal targets for practice and system improvements. These overlaps include support from providers, access to information, and regional and systemic gaps in services.

#### 5.1.1 Support From Providers

At the social network level, recommendations that focus on HCT preparation, involvement of primary care providers and care continuity are well established in the literature [2, 28, 53, 54, 55]. Not only does our study echo these recommendations, but it also reveals the disconnect between research, guidelines and care as experienced by families, suggesting inconsistent implementation into practice. It is well known that health care providers face many barriers to guideline implementation, such as inadequate training and the absence of formal responsibilities related to HCT [56]. As such, future research and policy work should focus on addressing these barriers. Potential interventions include health care provider training in complex care management [55, 57]; HCT (e.g., principles, interprofessional collaboration and transition interventions) [26, 58]; and recognizing, confronting and dismantling ableism [59, 60]. Clear outcome indicators are also needed to incentivize and drive practice change on a larger scale [61, 62].

Our study also emphasizes the important role of health care providers as advocates. While research on HCT often targets youth and caregiver self‐advocacy [12, 26, 63, 64, 65, 66], less attention has been given to health care provider advocacy [2, 3, 28, 67]. Furthermore, while numerous studies have documented the emotional strain that HCT places on youth and families [25, 31, 32, 34, 67, 68], recommendations and interventions focused on psychological support are notably lacking from the literature. As such, further work is needed to design and evaluate interventions that provide psychological support, such as peer support groups, which have the potential for reducing stress, empowering and increasing transition knowledge and planning for caregivers of YMC [69, 70].

#### 5.1.2 Access to Information

Another common concern of families in this study was the need for accessible and reliable information on the HCT process and adult services, a finding that is consistent with previous work [31, 68, 70, 71, 72]. Despite this being a known issue, families in this study accessed very few formal resources to support HCT. Since the completion of this study, the PCMCH has developed the CCKO (Complex Care for Kids Ontario) Youth Transition to Adult Care Toolkit to guide health care providers, youth and caregivers on navigating the HCT process [73]. While this resource is filling a clear need, families continue to seek information beyond the scope of this toolkit. Potential interventions to address family‐identified information gaps include a central resource for information on transition, tools to guide families through the HCT process, regional lists of services and providers, and provider roles dedicated to system navigation.

#### 5.1.3 Gaps in Services

At the societal level, family recommendations largely focused on improving the structure and availability of health care and community services for young adults with medical complexity. Their suggestions to implement payment models that better meet the longitudinal care needs of YMC are supported by recommendations from numerous Canadian and American medical and research authorities, including the Canadian Paediatric Society; the Society for Adolescent Medicine; and the American Academy of Pediatrics, among others [1, 2, 16, 55]. We reaffirm the urgent need for better health care payment models (e.g., that support care coordination) and the expansion of integrated primary care to support YMC across the lifespan. We encourage health care providers and decision makers to think creatively around system constraints and we support calls to work collaboratively to make these changes happen [74].

In addition to gaps in primary care, our study draws attention to essential social and community services that are missing from or lacking in the adult system, such as accessible day programmes, supportive housing and flexible options for home care. When services and programming were absent, caregivers became leaders, advocates and system builders. While families play an important role in advocacy and change, both study participants and our parent co‐researchers emphasized the need for health care providers and decision makers to partner with and lead efforts to expand access to these services.

Lastly, to ensure that future research, practice and policy changes have meaningful impact on the issues most important to families, it is critical people with lived experience are fully involved in the design and implementation of such initiatives. Most importantly, families need changes to be implemented urgently. The longer they wait for these essential services, the closer they will be pushed toward the point of crisis, which will not only create trauma for YMC and their families but also lead to negative system outcomes.

### 5.2 Limitations

Vast differences in the availability and delivery of health care and social services exist across regions, provinces and countries. As our study was limited to one Canadian province, findings may not be generalizable to other geographical regions. Additionally, participant eligibility was determined via self‐report, with the potential for inaccurate or imprecise identification of the intended population. However, it should be noted that while Cohen et al.'s framework is the most frequently used definition of YMC [18], it is difficult to operationalize, with existing algorithms facing significant limitations [55]. As such, the identification of YMC continues to be inconsistent across the literature. Furthermore, although multiple recruitment strategies were attempted, most of our participants were found through online parent communities. We acknowledge that those engaged in such communities may have distinct perspectives or experiences, and do not represent the experiences of all families of YMC. Lastly, our sample was limited to individuals who speak English, which excludes demographic groups that likely face even greater challenges with navigating the health and social care systems.

## 6 Conclusion

This qualitative case study explored HCT for YMC and their families, emphasizing the dynamic role of contextual factors, and family‐identified recommendations for practice and system change. Families urged health care providers and decision makers to act as advocates, provide psychological support and facilitate access to information. Our findings also draw attention to the need for more equitable and responsive health and social care systems and ongoing efforts to implement existing recommendations into practice.

## Author Contributions

**Lin Li:** conceptualization, data curation, formal analysis, funding acquisition, investigation, methodology, project administration, writing–original draft, writing–review and editing, visualization, resources. **Nancy Carter:** conceptualization, funding acquisition, methodology, supervision, writing–review and editing, resources. **Jan Willem Gorter:** conceptualization, funding acquisition, writing–review and editing, methodology, supervision, resources. **Linda Till:** funding acquisition, methodology, validation, writing–review and editing. **Marcy White:** funding acquisition, methodology, validation, writing–review and editing. **Patricia H. Strachan:** conceptualization, formal analysis, funding acquisition, methodology, supervision, writing–review and editing, resources.

## Ethics Statement

This study received ethical approval from the Hamilton Integrated Research Ethics Board under project number 11184.

## Consent

Informed consent was obtained electronically for all participants.

## Conflicts of Interest

The authors declare no conflicts of interest.

## Supporting information

Supporting information.

## Data Availability

The data that support the findings of this study are available on request from the corresponding author. The data are not publicly available due to privacy or ethical restrictions.
