# Supplementary material for: A Socioecological Approach to Support the Transition to Adult Care for Youth With Medical Complexity: Family Perspectives and Recommendations
Source: Health Expect. 2024 Dec 29;28(1):e70077. doi: 10.1111/hex.70077 (PMC11683189; doi:10.1111/hex.70077)
Supplement: Supplementary file 1 — Supporting information. [file HEX-28-e70077-s001.docx]

**Supplementary File 1: Consolidated Criteria for Reporting Qualitative Studies (COREQ) Checklist and Strategies to Enhance Rigour**

**Table S1.** COREQ Checklist. Details that are reported in the main text are indicated in blue. Details available in the published protocol are indicated in purple. Additional details only provided in this supplementary file are indicated in red.

| No. | Item | Guide Questions/Description | Reported/Additional Details |
| --- | --- | --- | --- |
| **Domain 1: research team and reflexivity** | | | |
| *Personal characteristics* | | | |
| 1 | Interviewer/ facilitator | Which author/s conducted the interview or focus group? | Primary author |
| 2 | Credentials | What were the researcher’s credentials? *E.g. PhD, MD* | Primary author: BScN and BFA degrees. Credentials of co-authors include PhD, RN, MD, MSW, MBA, and BA. |
| 3 | Occupation | What was their occupation at the time of the study? | Primary author: doctoral student and registered nurse. Co-authors: associate professor, professor, physician, parent with lived experience |
| 4 | Gender | Was the researcher male or female? | Primary author: female. Research team included male and female members. |
| 5 | Experience and training | What experience or training did the researcher have? | Primary author: graduate level research training in qualitative methods, experience as a pediatric registered nurse. Academic co-authors: senior researchers with previous experience conducting qualitative research, clinician experience. Parent co-researchers: TCPS2:CORE ethics training, ad hoc research training provided by primary author. |
| *Relationship with participants* | | | |
| 6 | Relationship established | Was a relationship established prior to study commencement? | No |
| 7 | Participant knowledge of the interviewer | What did the participants know about the researcher? *e.g. personal goals, reasons for doing the research* | Participants were told that the primary author (interviewer) was a nursing PhD student and registered nurse at a children’s hospital. The primary author explained that the research team is interested in learning more about the transition to adulthood and transfer to adult services for YMC and their families. |
| 8 | Interviewer characteristics | What characteristics were reported about the interviewer/facilitator? *e.g. Bias, assumptions, reasons and interests in the research topic* | The interviewer was a doctoral nursing student and registered nurse who worked in a children’s hospital, in an acute medicine and complex care setting. |
| **Domain 2: study design** | | | |
| *Theoretical framework* | | | |
| 9 | Methodological orientation and theory | What methodological orientation was stated to underpin the study? *e.g. grounded theory, discourse analysis, ethnography, phenomenology, content analysis* | Explanatory case study |
| *Participant selection* | | | |
| 10 | Sampling | How were participants selected? *e.g. purposive, convenience, consecutive, snowball* | Purposive strategies including criterion and maximum variation. Snowball sampling for additional family members and to facilitate access to hard-to-reach populations. |
| 11 | Method of approach | How were participants approached? *e.g. face-to-face, telephone, mail, email* | Social media recruitment. Potential participants contacted primary author by phone or email. |
| 12 | Sample size | How many participants were in the study? | 17 participants |
| 13 | Non-participation | How many people refused to participate or dropped out? Reasons? | 27 people responded to recruitment advertisements. 18 were eligible, 2 were ineligible, and 7 did not respond to follow-up attempts. One person declined to participate, stating that their transition experiences were too raw and difficult to talk about. 17 participants consented, and none dropped out. |
| *Setting* | | | |
| 14 | Setting of data collection | Where was the data collected? *e.g. home, clinic, workplace* | Virtual |
| 15 | Presence of non-participants | Was anyone else present besides the participants and researchers? | No |
| 16 | Description of sample | What are the important characteristics of the sample? *e.g. demographic data, date* | Reported in main text under “Findings: Participants” |
| *Data collection* | | | |
| 17 | Interview guide | Were questions, prompts, guides provided by the authors? Was it pilot tested? | Interview guide is available with the published protocol, and was pilot tested by a parent co-researcher. |
| 18 | Repeat interviews | Were repeat interviews carried out? If yes, how many? | 4 repeat interviews were carried out |
| 19 | Audio/visual recording | Did the research use audio or visual recording to collect the data? | Zoom videoconferencing software |
| 20 | Field notes | Were field notes made during and/or after the interview or focus group? | Field notes were recorded during and after each interview. |
| 21 | Duration | What was the duration of the interviews or focus group? | 30-100 minutes |
| 22 | Data saturation | Was data saturation discussed? | No. However, as per case study recommendations, data collection continued until enough data were gathered to: 1) support the main topics under study by two or more sources, and 2) adequately consider major rival explanations. |
| 23 | Transcripts returned | Were transcripts returned to participants for commend and/or correction? | No |
| **Domain 3: analysis and findings** | | | |
| *Data analysis* | | | |
| 24 | Number of data coders | How many data coders coded the data? | Two |
| 25 | Description of the coding tree | Did authors provide a description of the coding tree? | No. Data were initially coded inductively, meaning the coding tree was fluid. Subsequently, deductive analysis occurred by comparing the findings to the initial theoretical propositions. |
| 26 | Derivation of themes | Were themes identified in advance or derived from the data? | Themes were derived from the data (inductive analysis), but organized based on *a priori* conceptual frameworks |
| 27 | Software | What software, if applicable, was used to manage the data? | NVivo 12 |
| 28 | Participant checking | Did participants provide feedback on the findings? | Yes |
| *Reporting* | | | |
| 29 | Quotations presented | Were participant quotations presented to illustrate the themes / findings? Was each quotation identified? *e.g. participant number* | Yes |
| 30 | Data and findings consistent | Was there consistency between the data presented and the findings? | Yes |
| 31 | Clarity of major themes | Were major themes clearly presented in the findings? | Yes |
| 32 | Clarity of minor themes | Is there a description of diverse cases or discussion of minor themes? | Yes, subcategories are described and variations within those subcategories (e.g., the impact of nuances in context). |

Checklist from: Tong A, Sainsbury P, Craig J. Consolidated criteria for reporting qualitative research (COREQ): a 32-item checklist for interviews and focus groups. *International Journal for Quality in Health Care*. 2007;19(6):349-357. doi:[10.1093/intqhc/mzm042](https://doi.org/10.1093/intqhc/mzm042)

**Table S2.** Case Study Strategies to Enhance Rigour

| Criteria | Strategies Employed |
| --- | --- |
| Construct validity | - Multiple data sources were converged (participants, data types) - Key concepts were clearly defined in the study protocol - A conceptual framework and theoretical propositions were used to link the research questions to the findings - Member checking was performed by seeking participant feedback on researchers’ interpretations - Peer auditing was done by the research team including parent co-researchers |
| Internal validity | - Data analysis techniques of pattern matching and explanation building were used - Rival explanations were addressed during analysis - Peer auditing |
| External validity | - *A priori* theory and study propositions were used to inform the research methods |
| Reliability | - Methods were described in detail in a case study protocol - Data were managed systematically in a database - Chain of evidence was maintained by linking the findings to the original study propositions |
